# Proteomic Analysis of the Antibacterial Effect of Improved Dian Dao San against *Propionibacterium acnes*

**DOI:** 10.1155/2022/3855702

**Published:** 2022-02-09

**Authors:** Shuaijun Ren, Wenjiao Wang, Min Jia, Lili An, Ting Tang, Xin Liu

**Affiliations:** ^1^Guizhou University of Traditional Chinese Medicine, No. 50, Shidong Rd., Nanming District, Guiyang City, Guizhou Province 550001, China; ^2^Dermatology Department, The First Affiliated Hospital of Guizhou University of Traditional Chinese Medicine, No. 71, Baoshan North Rd., Yunyan District, Guiyang City, Guizhou Province 550002, China; ^3^Ethnic Medicine, Qiannan Traditional Chinese Medicine Hospital, No. 32 Jianjiang Middle Rd., Duyun City, Guizhou Province 558000, China

## Abstract

*Propionibacterium acnes (P. acnes)* is a major pathogen of acne vulgaris. The traditional Chinese medicine (TCM) compound prescription, Dian Dao San (DDS), is effective for treating *P. acnes*. Previous clinical work by our team demonstrated that improved Dian Dao San (IDDS) has better antibacterial effects. However, the mechanism of IDDS inhibition of *P. acnes* is still unknown. Hence, the isobaric tags for relative and absolute quantitation (iTRAQ) technology was applied to explore the antibacterial mechanism of IDDS against *P. acnes.* Our results suggested that the antibacterial mechanism of IDDS was related to the glycolytic pathway. gap, pgk, and tpiA enzymes were found to be potential target proteins in the bacterial glycolytic pathway as an antibacterial mechanism of inhibition. In addition, SEM and TEM analyses revealed that IDDS may destruct bacterial plasma membrane and cell wall. The results provide a reliable, direct, and scientific theoretical basis for wide application of IDDS.

## 1. Introduction

Acne vulgaris, caused by proliferation of Gram-positive and anaerobic *Propionibacterium acnes* (*P. acnes*), is a pilosebaceous inflammatory skin disease affecting 90% of teenagers globally and persisting or recurring in adulthood [1, 2]. In recent years, the prevalence of *P. acnes*-derived acne vulgaris has increased, with dire implications on patients' cosmetic features [3, 4]. Therefore, necessary measures are needed to control the increasing incidence of *P. acnes*-derived acne vulgaris.

Clinically, acne vulgaris caused by *P. acne* is usually treated with antibiotics, retinoic acid, and hormonal drugs [5]. These drugs have varying degrees of side effects, ranging from skin irritation to teratogenicity [6]. Moreover, *P. acnes* exhibits multidrug resistance due to antibiotic overuse or abuse [7]. Therefore, seeking safer and more effective treatment options is necessary.

Medicines used in traditional Chinese medicine (TCM) have broad-spectrum antibacterial activities and their antibacterial mechanisms exhibit multitarget effects [8]. Additionally, some TCM compounds and monomer prescriptions were shown to inhibit the growth of bacteria, virulence factor activity, and related gene expression [9].

Dian Dao San (DDS), a prescription of topical powder from Wu Qian's The Golden Mirror of Medicine in the Qing Dynasty, is composed of rhubarb and sulfur. It has been used clinically to treat acne vulgaris [10]. This prescription with definite curative effect was recommended as an external medicine in the guidelines for diagnosis and treatment of acne vulgaris in 2019 in China [1]. According to previous studies, DDS exerts its function by attenuating inflammatory responses and reducing hyperkeratosis. In those studies, we found that higher proportions of DDS formulas (the ratio of rhubarb and sulfur from 1 : 1 to 2 : 1) have better anti-*P. acnes*-derived acne vulgaris effects. However, the mechanisms underlying improved DDS (IDDS)'s effect on *P. acnes* are poorly understood.

Due to its good reproducibility, accuracy, and high-throughput characteristics, proteomics is one of the methods applied in the study of TCM compound prescriptions [11, 12]. It is highly compatible with the multitarget and multipathway regulatory features of TCM [13]. Studying TCM prescriptions using proteomics can help reveal the whole proteomic changes involved in multiple channels and targets after the action of TCM prescriptions. This approach will provide a more comprehensive understanding of their mechanisms of action at the molecular level [14].

In this study, the isobaric tags for relative and absolute quantitation (iTRAQ) technology was used to demonstrate the antibacterial mechanism of IDDS against *P. acnes.* The results showed that the antibacterial mechanism of IDDS was related to the glycolytic pathway, plasma membrane, and cell wall. To verify the accuracy of our findings, glyceraldehyde-3-phosphate dehydrogenase (gap), phosphoglycerate kinase (pgk), glucokinase, and triosephosphate isomerase (tpiA), which are proteins in the glycolytic pathway, were selected for evaluation at the mRNA level. Furthermore, scanning electron microscope (SEM) and transmission electron microscope (TEM) analyses confirmed the correlation between the antibacterial mechanism of IDDS and the plasma membrane and cell wall. This study provided relevant theoretical and experimental bases for the clinical application of IDDS.

## 2. Materials and Methods

### 2.1. Medicinal Materials and Strains

The TCM medicinal prescription (rhubarb and sulfur were obtained from The First Affiliated Hospital of Guizhou University of Traditional Chinese Medicine) used in the experiment was an ingredient from The Golden Mirror of Medicine. *P. acnes* (ATCC11827) samples were purchased from Shanghai Zhichenhui Biotechnology Company Limited (Shanghai, China).

### 2.2. IDDS Preparation

First, 30 g of glycerin, as a solvent, was mixed with 30 g of sulfur powder. Afterwards, 60 g of rhubarb powder was added to a soaking-paper to make IDDS (rhubarb : sulfur = 2 : 1). A volume of 360 mL of purified water was added to the resulting mixture. The solution was soaked for 30 min, decocted for 30 min, and filtered. After filtration, the filtrate was collected. An appropriate amount of water (about 180 mL) was added to the residue and boiled. The solution was left to boil at a low fire for 15 min. After boiling, the resulting filtrate was collected. Finally, the two filtrates were diluted to a volume of 90 mL to obtain a medicinal solution of 1 g/mL concentration.

### 2.3. Culture Preparation of Strain and Determination of the Minimal Inhibitory Concentration (MIC) of IDDS

The MIC of IDDS against *P. acnes* (ATCC11827) was determined by serial dilution. Briefly, *P. acnes* was cultured in brain heart infusion (BHI) broth (Difco, Hangzhou, China) under anaerobic conditions, created using an anaerobic bag at 37°C for 48 h. The bacterial suspension was adjusted with a culture concentration of approximately 1 × 10^6^ colony-forming units (CFU)/mL [15]. Finally, 100 mL of IDDS was added to the 96-well plates to dilute the bacterial suspension serially. Concurrently, the cultures were grouped into the prescription color control group, bacterial culture control group, and medium group. The MIC was defined as the lowest concentration of *P. acnes* that visually inhibited growth, with triplicates in each experiment [16].

### 2.4. iTRAQ and Bioinformatics Analysis

Proteins were extracted from the treated (using IDDS) and untreated cultures of *P. acne* with 1/2 MIC (31.25 mg/mL). iTRAQ analysis was performed at Hangzhou Lianchuan Biotechnology Co., Ltd. (Hangzhou, China). Using quantitative proteomics analysis, the effect of 1/2 MIC IDDS (31.25 mg/mL) on *P. acnes* was studied. Isobaric reagents were used in the analysis of bacterial cultures treated with or without IDDS. After the samples were combined, SCX chromatography was used to separate the mixture for liquid chromatography (LC) and mass spectrometry/mass spectrometry (MS/MS) analyses. Bioinformatics analysis (Blast2Go), as mentioned before, was used to annotate the identified proteins according to their biological functions. They were further classified as altered proteins using the KEGG pathway. Finally, STRING, used for mapping the protein interaction network, was used to significantly analyze differently expressed proteins.

### 2.5. qPCR Analysis

Total RNA from bacterial samples (three biological replicas per sample) were isolated using Trizol reagent (Ambion Inc., Texas, America), and cDNA was reverse-transcribed from each RNA using the reverse transcription kit (Vazyme Biotech Co., Ltd., Nanjing, China). With the 16S RNA expression level serving as an internal reference, qPCR was used to validate RNA expression of *gap*, *pgk*, *tpiA*, and *glucokinase*. The primers were synthesized by Wuhan Biofavor Biotech Service Co., Ltd. (Wuhan, China) and are listed in Table 1. The PCR was performed with the following reagents: 4 *µ*l of cDNA, 10 *µ*l of SYBR Green Master Mix (Vazyme Biotech Co., Ltd., Nanjing, China), 0.4 *µ*l of 50 × ROX Reference Dye 2, 0.4 *µ*l of each primer (10 *μ*M), and 4.8 *µ*l of H_2_O. The PCR proceeded under the following conditions: 95°C predenaturation for 10 min, followed by 40 cycles at 95°C denaturation for 15 s, and annealing and extension at 60°C for 60 s. A melt curve was plotted for 95°C at 15 s, 60°C at 60 s, and 95°C at 15 s. The experiments were performed in triplicate.

### 2.6. SEM and TEM Analyses

The bacterial culture and treatment methods were performed, as described above. The bacterial suspension was collected after 3 h and centrifuged; the supernatant was discarded. Thereafter, the precipitate was collected after it was gently washed thrice with PBS. SEM samples were prepared by dehydration with gradient alcohol, lyophilization, and gold spraying, before they were sealed into slides. The TEM samples were obtained by following the negative staining procedure. The samples were scanned by Chengdu Lilai Biotechnology Co., Ltd. (Chengdu, China).

## 3. Results and Discussion

### 3.1. Proteomics Analysis of the Differential Expression after Treatment with IDDS

**Figure 1:**
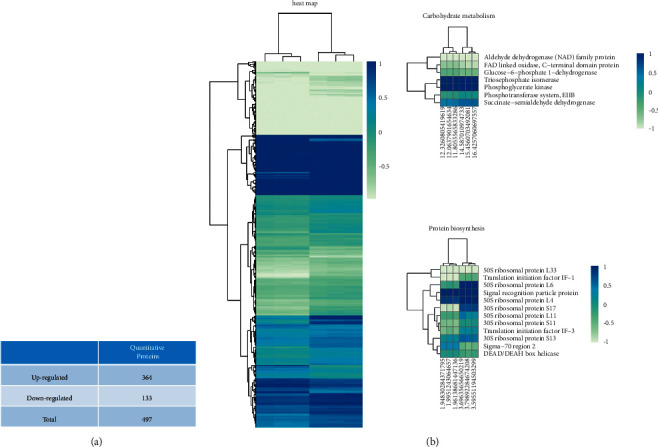
Differential expression of proteins in *P. acnes* under 31.25 mg/ml by iTRAQ. (a) The number of altered proteins. (b) 497 differentially expressed proteins are illustrated with a heat map.

A total of 1,610 proteins were identified; 497 proteins showed significant differences in expression between the treated (using IDDS) and untreated *P. acnes* cultures, based on a fold change (>1.2 or *P* < 0.05). The comparison between the groups revealed that, compared with proteins in the untreated group, 364 proteins in the treated group were upregulated and 133 proteins were downregulated (Figure 1(a)). The differentially expressed proteins are illustrated in the heat map (Figure 1(b)).

### 3.2. Analysis of Altered Proteins after IDDS Treatment Using the GO Annotation and KEGG Pathway

**Figure 2:**
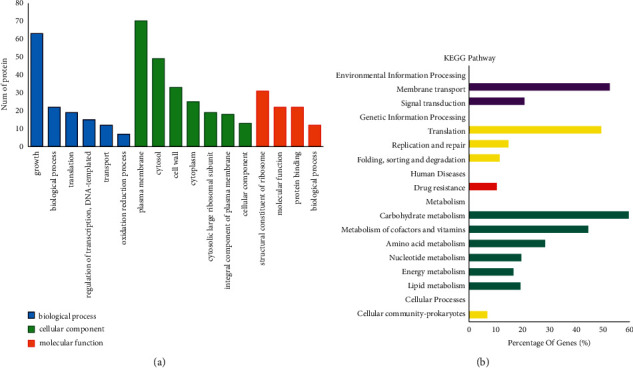
GO annotations and KEGG pathways: (a) cellular component, biological process, and molecular function; (b) main KEGG pathway.

Bioinformatics analyses of the differential proteins were performed to observe the enrichment characteristics of their overall functions. First, GO ontology is a comprehensive cross-species database, mainly divided into biological process, molecular function, and cellular component. Each protein had a specific term for its description [17]. By GO annotation (Figure 2(a)), most proteins were mainly enriched in growth, plasma membrane, cytosol, cell wall, and structural constituents of ribosome. This suggests that the antibacterial mechanism of IDDS may be related to growth and possibly acts by destroying the plasma membrane and cell wall and interfering with the structural constituents of ribosomes. Moreover, the exercise of biological functions requires the coordination of proteins, and pathway analysis could help in comprehensive and systematic understanding of antibacterial mechanisms of IDDS [18]. Therefore, according to KEGG pathway analysis, among these enriched proteins, proteins for carbohydrate metabolism had the highest number, suggesting that this pathway was significantly affected. This finding suggests that IDDS may mainly affect the carbohydrate metabolism pathway to exert its antibacterial mechanism (Figure 2(b)).

### 3.3. Differential Protein-Protein Interaction (PPI) Network Analysis

**Figure 3:**
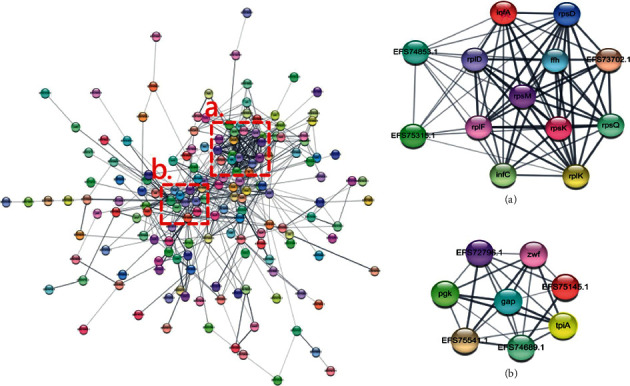
STRING network of significantly differential proteins of *P. acne* under the action of 31.25 mg/mL IDDS. The interactions between different types of proteins are represented by lines. (a) Protein biosynthesis-related proteins. (b) Carbohydrate metabolism-related proteins.

To explore which proteins were more relevant to the antibacterial mechanism of IDDS, PPI was used to analyze the differential proteins. Among 497 proteins, 198 proteins were displayed in the PPI network. As shown in Figure 3, two protein clusters were formed, but further analysis of the data is needed to find key proteins. In screening the proteins, the number of lines between proteins are counted. A high number of lines indicates a close interactive relationship, which was screened as possible key proteins [19]. According to this method, 21 latent target proteins were selected from these two located in Figures 3(a) and 3(b). Thereafter, these proteins were separately mapped using STRING.

In Figure 3(a), infA and infC were translation initiation factor proteins, which play an essential role in regulating initial protein synthesis [20]. Translation initiation factor proteins promote the combination of 50 S ribosomal units with 30 S ribosomes into the 30 S initiation complex to initiate the DNA synthesis [21]. In addition to KEGG pathway analysis, this cluster included mainly translation-related 50 S ribosomal and 30 S ribosomal proteins. Ribosomes are the sites for protein synthesis, and translation plays a highly coordinated role in the complex biosynthesis of proteins [22]. Therefore, we infer that the protein synthesis pathway was affected. From the analysis, we found that relevant protein biosynthesis-related ribosomal proteins showed upregulated expression. Protein synthesis is an important step in stress response [23]. We surmise that *P. acne* had a stress response at sub-MIC. This response was usually a self-defense mechanism, which allowed the bacteria to overcome changes in the surrounding environment, contributing to bacterial survival [24]. Therefore, the antibacterial mechanism of IDDS can be concluded to be independent of the protein synthesis pathway.

According to the bioinformatics analysis, another cluster relevant to carbohydrate metabolism was also significantly altered (Figure 3(b)). *gap*, *pgk*, and *tpiA* participate in carbohydrate metabolism through glycolysis [25]. *tpiA* is responsible for efficient energy production, catalyzing reversible interconversion glyceraldehyde-3-phosphate (G3P) and dihydroxyacetone phosphate (DHAP) [26, 27]. Central to the glycolytic pathway, *gap* catalyzes G3P into 1,3-bisphosphoglycerate (1,3 BPG), a high-energy compound [28, 29]. Thereafter, the product is catalyzed by *pgk* to produce glycerate 3-phosphate (3-PG) and ATP [30]. *pgk* is the first regulator of ATP production and plays an important role in energy production [31]. Based on this pathway, proteins were highly distributed in the glycolytic pathway, which affected energy production. This finding implies that the glycolytic pathway may be responsible for the antibacterial mechanism of IDDS. In addition, inferring from the analysis results, the antibacterial mechanism of IDDS may also affect the plasma membrane and cell wall.

### 3.4. Inhibition of the Glycolytic Pathway

**Figure 4:**
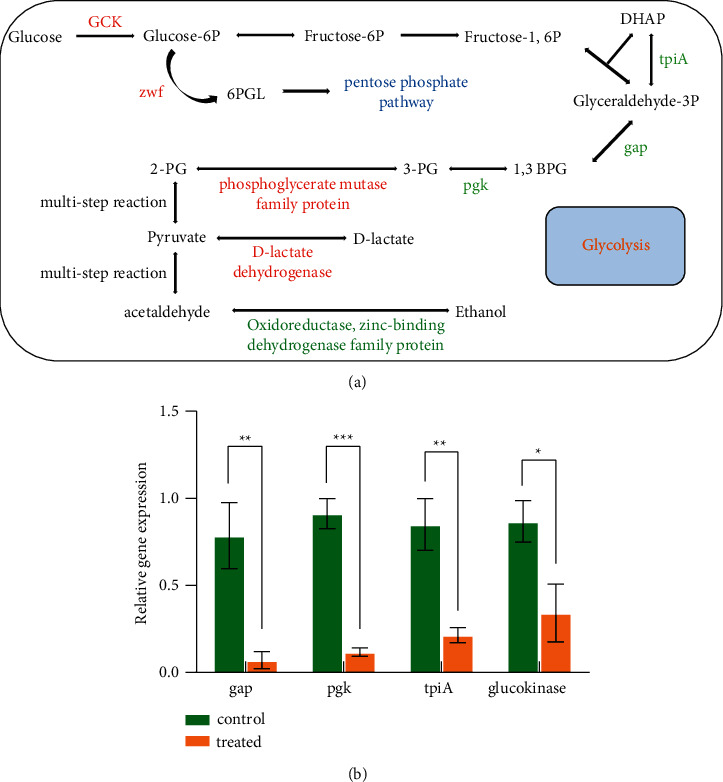
(a) Pathway of carbohydrate metabolism (pathways derived from the KEGG database). Expressed proteins with red fonts, indicating upregulation, and green fonts, indicating downregulation. (b) Relative gene expression evaluation of gap, pgk, tpiA, and glucokinase by qPCR. Significant differences are represented by asterisks (*∗*).

In this study, we further analyzed important proteins that play a role in the glycolytic pathway, and found that the expression of *gap*, *pgk*, and *tpiA* was downregulated, whereas glucokinase was upregulated. Glucokinase catalyzes an irreversible reaction of glucose to glucose-6-phosphate (glucose-6P), which controls the key step for glycolysis [32, 33]. Therefore, upregulation of the glucokinase protein may initiate glycolysis and catalyze the production of glucose-6P. At this stage, glucose-6P lies at the beginning of two primary metabolic pathways: one branch of glycolysis towards the pentose phosphate pathway (PPP) by 6-phosphogluconolactonase (zwf) catalysis; the other branch continues through the glycolytic pathway [34, 35]. The upregulation of zwf expression means a shift of glucose-6P towards PPP, whereas less glucose-6P enters the glycolytic pathway, leading to the downregulated expression of *pgk*, *gap*, and *tpiA* [36]. The insufficient catalysis of glucokinase causes the glucose transporter to reduce glucose entry, thereby interfering with the catalysis of key enzymes [37]. Furthermore, D-lactate dehydrogenase level was elevated after multiple steps, which easily causes the accumulation of lactic acid, further inhibiting bacterial growth and producing toxic effects [38]. In summary, reduced energy supply leads to energy deficiency and growth inhibition in bacteria [39]. IDDS exerts its antibacterial mechanism by inhibiting the glycolytic pathway and targeting gap, pgk, and tpiA enzymes as potential target proteins, as shown in Figure 4(a).

To verify this conclusion, quantitative qPCR was used to evaluate the mRNA levels of these proteins. The gene expression levels of gap, pgk, and tpiA were consistent with their protein expression, whereas mRNA levels for expression of glucokinase had reduced, as expected (Figure 4(b)). These findings provide protein targets for subsequent development of new antibacterial drugs.

### 3.5. Effect on the Plasma Membrane and Cell Wall

**Figure 5:**
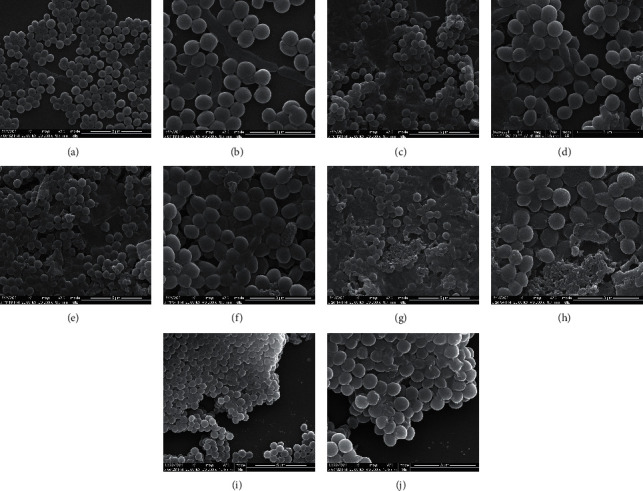
Observation of the inhibitory effect of IDDS on *P. acnes* for 3 h by SEM. (a) Untreated *P. acnes* group (20000x); (b) Untreated *P. acnes* group (40000x); (c) 62.5 mg/mL IDDS treatment group (20000x); (d) 62.5 mg/mL IDDS treatment group (40000x); (e) 125 mg/mL IDDS treatment group (20000x); (f) 125 mg/mL IDDS treatment group (40000x); (g) 250 mg/mL IDDS treatment group (20000x); (h) 250 mg/mL IDDS treatment group (40000x); (i) Rhubarb treatment group (20000x); (j) Rhubarb treatment group (40000x).

The GO annotation revealed that IDDS likely affects the plasma membrane and cell wall. Therefore, SEM analysis was used to assess the changes in morphology of *P. acne* after treatment. The results are shown in Figure 5.

In the untreated *P. acne* group, the bacteria were arranged neatly and orderly. The cells were compact with intact plasma membranes and smooth cell walls (Figures 5(a)-5(b)). After treatment with IDDS (MIC, 62.5 mg/mL), some changes in bacterial surface morphology were observed. The cells appeared slightly shrunk and disorderly arranged. Moreover, cellular contents had leaked and dissolved the cells into clumps (Figures 5(c)-5(d)). Higher drug concentrations (125 mg/mL of IDDS) caused an increase in the number of crumpled cells and a more proportional appearance of fusion and adhesion (Figures 5(e)-5(f)). Upon further increase in drug concentration (250 mg/mL of IDDS), the following changes were observed: ambiguity of boundaries between cells, formation of granular features and vesicles on bacterial surface, considerable reduction in bacterial size, and formation of pores (Figures 5(g)-5(h)).

**Figure 6:**
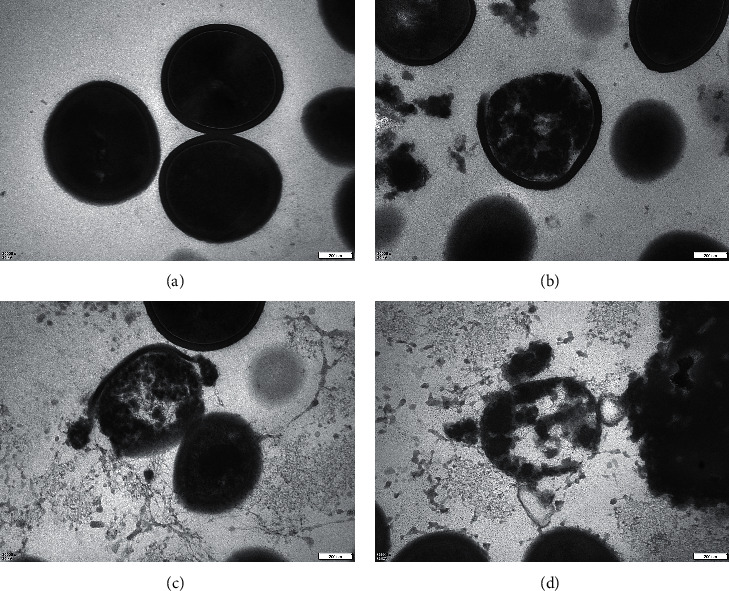
Observation of the inhibitory effect of IDDS on *P. acnes* for 3 h by TEM. (a) Untreated *P. acnes* group; (b) 62.5 mg/mL IDDS treatment group; (c) 125 mg/mL IDDS treatment group; (d) 250 mg/mL IDDS treatment group.

To further understand the internal ultrastructural alterations, TEM scanning was used to obtain high-magnification images of the cell wall and plasma membrane (Figure 6). We observed, as shown in Figures 6(a)-6(b), that the untreated *P. acne* group had intact plasma membranes, regular and smooth cell wall surface, homogeneous contents in cytoplasm, and abundant ribosomes. The treated *P. acne* group, however, exhibited several deformations. Under the action of IDDS at MIC (62.5 mg/mL), some parts of the plasma membrane and cell wall were absent, and the cytoplasm appeared nonuniform (Figure 6(b)). At medium drug concentrations (125 mg/mL), the plasma membrane and cell wall were further deformed with leakage of cytoplasmic contents, containing cytoplasmic vacuoles (Figure 6(c)). Finally, the overall morphology of *P. acne* in the IDDS 4x MIC (250 mg/mL) treatment group appeared blurred with an incomplete structure. The cell wall and ruptured plasma membrane were completely lost with extravasated cytoplasmic contents (Figure 6(d)).

Sulfur has been known to be injurious to bacteria since the first half of the last century. The details of changes in morphology and membrane integrity were only recently uncovered [40]. Therefore, an experiment was conducted to assess the effect of rhubarb only on plasma membrane and cell wall. Figures 5(i)-5(j) show that rhubarb has a destructive effect on the structure of *P. acnes*, leading to shrinkage and content leakage. Therefore, the additive effect from the combination of rhubarb and sulfur may destroy the cell wall and cell membrane of *P. acnes*.

Therefore, SEM and TEM analyses confirmed the plasma membrane and cell wall as targets of the drug's action on *P. acne*. Our experimental results are consistent with findings in the literature, which report that TCM prescriptions affect the plasma membrane of *P. acne* by changing plasma membrane permeability, causing bacterial rupture and cytoplasmic spillage. Eventually, these effects lead to direct destruction of the cell structure and cell death [41].

## 4. Conclusions

IDDS is a Chinese herbal compound used to treat acne. *P. acnes* is the main pathogen of acne vulgaris. Our research explored the antibacterial mechanism of IDDS against *P. acnes.* iTRAQ and experimental verification proved that the glycolytic pathway was the primary antibacterial mechanism. Our findings indicated that gap, pgk, and tpiA may be potential drug antibacterial targets. In addition, the cell membrane and cell wall were involved in the antibacterial mechanism underlying IDDS action against *P. acnes.* These experimental results provide a basis for the clinical application of IDDS.

## Figures and Tables

**Table 1:** Primer sequence.

Gene	Primer	Sequence (5′–3′)
16S RNA	Forward	GGACGAGTTATCCTGGCG
	Reverse	GCAGACTCGTTGCCTTCA

gap	Forward	GCTTGCTGGCGACGAG
	Reverse	GAAGGCTGCCGAGGGT

pgk	Forward	TCGGTGACTCCCTACTTGA
	Reverse	TCGGAAGGAATCTCGTCAG

tpiA	Forward	CACAACGAGTCCGACGAAC
	Reverse	CACAGCAGATGATTGGGGT

Glucokinase	Forward	GCACTCGTTATCAATGGGCG
	Reverse	ATGAGGTTATCGGGATTGCG

## Data Availability

The data used to support the findings of this study are available from the corresponding author upon request.

## References

[1] Sun K. L., Chang J. M. (2017). Special types of folliculitis which should be differentiated from acne. *Dermato-Endocrinology*.

[2] Tan A. U., Schlosser B. J., Paller A. S. (2017). A review of diagnosis and treatment of acne in adult female patients. *International Journal of Women’s Dermatology*.

[3] Muthupalaniappen L., Tan H. C, Puah J. W (2014). Acne prevalence, severity and risk factors among medical students in Malaysia. *La Clinica Terapeutica*.

[4] Kim K.-H., Lee S.-C., Park Y.-B., Park Y.-J. (2017). Cardiff Acne Disability Index: cross-cultural translation in Korean and its relationship with clinical acne severity, pathological patterns, and general quality of life. *Journal of Traditional Chinese Medicine*.

[5] Zhang T. B., Bai Y. P. (2019). Research progress in the treatment of acne vulgaris. *Chinese Journal of Integrated Traditional Chinese and Western Medicine Dermatology and Venereology*.

[6] Ju Q. (2018). Application of tretinoin drugs in the treatment of acne. *Skin diseases and venereal diseases*.

[7] You Z. P., Cao B. L. (2018). Research progress on antibiotic resistance of propionibacter acnes. *Chinese Journal of Dermatovenereology*.

[8] Wei Q. X. (2019). Research progress of tanreqing treating multi-drug-resistant bacteria infection. *Journal of Clinical Rational Use*.

[9] Li Z., Chen W., Hu J., Yin H., Peng Y., Hu X. (2019). Research progress on antibacterial status and mechanism of traditional Chinese medicine against Candida. *Shanghai Journal of Traditional Chinese Medicine*.

[10] Yang L. (2018). Research and creation of new prescriptions for treating acne. *Journal of Hunan University of Traditional Chinese Medicine*.

[11] Le L., Jiang B., Xu J., Hu K., Chen S. (2016). TCM proteomics research strategy. *Chinese Journal of Traditional Chinese Medicine*.

[12] Xie P., Liu B., Chen R., Yang B., Dong J., Rong L. (2014). Comparative analysis of serum proteomes: identification of proteins associated with sciatica due to lumbar intervertebral disc herniation. *Biomedical reports*.

[13] Sun S., Du Y., Li S. (2021). Anti‑inflammatory activity of different isolated sites of Chloranthus serratus in complete Freund’s adjuvant‑induced arthritic rats. *Experimental and Therapeutic Medicine*.

[14] Enany S., Ato M., Matsumoto S. (2021). Differential protein expression in exponential and stationary growth phases of *Mycobacterium avium* subsp. hominissuis 104. *Molecules*.

[15] Huang W.-C., Tsai T.-H., Chuang L.-T., Li Y.-Y., Zouboulis C.-C., Tsai P.-J. (2013). Anti-bacterial and anti-inflammatory properties of capric acid against Propionibacterium acnes: a comparative study with lauric acid. *Journal of Dermatological Science*.

[16] Barlow V. L., Lai S.-J., Chen C.-Y., Tsai C.-H., Wu S.-H., Tsai Y.-H. (2020). Effect of membrane fusion protein AdeT1 on the antimicrobial resistance of *Escherichia coli*. *Scientific Reports*.

[17] Ashburner M., Ball C. A., Blake J. A. (2000). Gene Ontology: tool for the unification of biology. *Nature Genetics*.

[18] Liu X., Wang J., Chen M. (2019). Comparative proteomic analysis reveals drug resistance of Staphylococcus xylosus ATCC700404 under tylosin stress. *BMC Veterinary Research*.

[19] Li M., Zheng R., Zhang H., Wang J., Pan Y. (2014). Effective identification of essential proteins based on priori knowledge, network topology and gene expressions. *Methods*.

[20] Lapidot M., Karniel U., Gelbart D. (2015). A novel route controlling begomovirus resistance by the messenger RNA surveillance factor pelota. *PLoS Genetics*.

[21] Madison K. E., Abdelmeguid M. R., Jones-Foster E. N., Nakai H. (2012). A new role for translation initiation factor 2 in maintaining genome integrity. *PLoS Genetics*.

[22] Harish A., Caetano-Anollés G. (2012). Ribosomal history reveals origins of modern protein synthesis. *PLoS One*.

[23] Liu P., Zhang H., Yu B., Xiong L., Xia Y. (2015). Proteomic identification of early salicylate- and flg22-responsive redox-sensitive proteins in Arabidopsis. *Scientific Reports*.

[24] Ryan D., Pati N. B., Ojha U. K. (2015). Global transcriptome and mutagenic analyses of the acid tolerance response of *Salmonella enterica* serovar Typhimurium. *Applied and Environmental Microbiology*.

[25] Ming T., Han J., Li Y. (2018). A metabolomics and proteomics study of the Lactobacillus plantarum in the grass carp fermentation. *BMC Microbiology*.

[26] Karmakar S., Datta K., Molla K. A. (2019). Proteo-metabolomic investigation of transgenic rice unravels metabolic alterations and accumulation of novel proteins potentially involved in defence against Rhizoctonia solani. *Scientific Reports*.

[27] Panyushkina A., Matyushkina D., Pobeguts O. (2020). Understanding stress response to high-arsenic gold-bearing sulfide concentrate in extremely metal-resistant acidophile sulfobacillus thermotolerans. *Microorganisms*.

[28] Mandic R., Agaimy A., Pinto-Quintero D. (2020). Aberrant expression of glyceraldehyde-3-phosphate dehydrogenase (GAPDH) in warthin tumors. *Cancers*.

[29] Wang X., Sakata K., Komatsu S. (2018). An integrated approach of proteomics and computational genetic modification effectiveness analysis to uncover the mechanisms of flood tolerance in soybeans. *International Journal of Molecular Sciences*.

[30] Zhang Y., Cai H., Liao Y., Zhu Y., Wang F., Hou J. (2020). Activation of PGK1 under hypoxic conditions promotes glycolysis and increases stem cell‑like properties and the epithelial‑mesenchymal transition in oral squamous cell carcinoma cells via the AKT signalling pathway. *International Journal of Oncology*.

[31] Zheng Y.-X., Zhang X.-X., Hernandez J. A. (2019). Transcriptomic analysis of reproductive damage in the epididymis of male Kunming mice induced by chronic infection of Toxoplasma gondii PRU strain. *Parasites & Vectors*.

[32] Farrugia F., Aquilina A., Vassallo J., Pace N. P. (2021). Bisphenol A and type 2 diabetes mellitus: a review of epidemiologic, functional, and early life factors. *International Journal of Environmental Research and Public Health*.

[33] Lai T.-C., Hu M.-C. (2019). Regulation of liver receptor homologue-1 by DDB2 E3 ligase activity is critical for hepatic glucose metabolism. *Scientific Reports*.

[34] Jiang Y., Jiao H., Sun P., Yin F., Tang B. (2020). Metabolic response of Scapharca subcrenata to heat stress using GC/MS-based metabolomics. *Peer Journal*.

[35] Oates J. R., McKell M. C., Moreno-Fernandez M. E. (2019). Macrophage function in the pathogenesis of non-alcoholic fatty liver disease: the Mac Attack. *Frontiers in Immunology*.

[36] Jia F. F. (2009). *Analysis of Bacillus Subtilis Proteome Based on Two-Dimensional Gel Electrophoresis and Mass Spectrometry*.

[37] Zheng X., Boyer L, Jin M (2016). Metabolic reprogramming during neuronal differentiation from aerobic glycolysis to neuronal oxidative phosphorylation. *Elife*.

[38] Billig S., Schneefeld M., Huber C., Grassl G. A., Eisenreich W., Bange F.-C. (2017). Lactate oxidation facilitates growth of *Mycobacterium tuberculosis* in human macrophages. *Scientific Reports*.

[39] Yu B., Sun W., Huang Z. (2021). Large-scale preparation of highly stable recombinant human acidic fibroblast growth factor in *Escherichia coli* BL21(DE3) plysS strain. *Frontiers in Bioengineering and Biotechnology*.

[40] Saedi S., Shokri M., Rhim J.-W. (2020). Antimicrobial activity of sulfur nanoparticles: effect of preparation methods. *Arabian Journal of Chemistry*.

[41] Zhang M. M. (2015). *Study on the Preparation Technology and Antibacterial Activity of Ginkgolic Aci*.

